# Effectiveness of Dynamic Brace in Posterior Tibial Translation in Acute PCL Lesion: A Pilot, Single Center Exploratory Study

**DOI:** 10.3390/healthcare14070953

**Published:** 2026-04-05

**Authors:** Giorgio Zappalà, Michelangelo Delmedico, Davide Ciclamini, Nicholas Trapella, Carlo Pasquali, Camilla Crespi, Mario Ronga

**Affiliations:** 1 ASST Papa Giovanni XXIII, 24127 Bergamo, Italy; giorgio.zappala@libero.it (G.Z.); michelangelo.del@gmail.com (M.D.); 2 Orthopaedic and Trauma Operative Unit, Department of Health Sciences, University of Eastern Piedmont, Via Paolo Solaroli, 17, 28100 Novara, Italy; davide.ciclamini@uniupo.it (D.C.); nicholas.trapella@gmail.com (N.T.); carlo.pasquali95@gmail.com (C.P.); camillacrespi99@gmail.com (C.C.)

**Keywords:** posterior cruciate ligament, PCL injury, conservative treatment, dynamic brace, knee instability

## Abstract

**Background**: Acute posterior cruciate ligament (PCL) injuries are uncommon and often challenging to treat. While conservative treatment is frequently proposed in the acute phase, conventional rigid bracing may lead to complications such as joint stiffness and quadriceps atrophy. Dynamic braces applying posterior to anterior force during flexion have been proposed as a more functional alternative. **Purpose:** To evaluate the biomechanical efficacy of a dynamic PCL brace in reducing posterior tibial translation during the acute post-traumatic phase using standardized stress radiographs. **Methods**: The study was conducted on 11 patients with acute PCL injuries (four isolated, seven multiligamentous) treated within 15 days from trauma. Posterior tibial translation was measured with X-rays at 90° of flexion under four conditions: static (resting), stress (150 N), brace unloaded, and brace loaded (50 N posterior force). Three blinded orthopedic surgeons performed all measurements independently. **Results:** The dynamic brace significantly reduced posterior tibial translation across all conditions. Translation under stress was reduced from a mean of 7.1 mm to 2.68 mm with the loaded brace (*p* < 0.001). **Conclusions:** The study demonstrates that dynamic bracing provides effective biomechanical control of posterior tibial translation in the acute PCL injury. These findings support the potential role of dynamic bracing in conservative treatment protocols.

## 1. Introduction

Posterior cruciate ligament (PCL) injuries are uncommon, with an estimated incidence of 1.8–2 per 100,000 individuals per year [1,2] and isolated PCL tears, occurring without concomitant ligamentous lesions, are rare: 1% of all acute knee injuries [3]. These lesions most commonly result from direct anterior to posterior forces applied to the proximal tibia, typically sustained in motor vehicle accidents or high-impact sports [1,4].

The PCL has demonstrated considerable potential for spontaneous healing, largely due to its rich vascular supply; however, the optimal management of acute injuries remains controversial [5]. Several studies have shown clinically relevant patient-reported improvements after conservative treatment [6]. Good outcomes were achieved in the short- and medium-term follow-up [7,8], with a successful return to sports activities even in high demanding athletes [2]. However, suboptimal long-term results have been reported especially in terms of residual instability, increased risk of meniscal lesions, early development of arthritis and posterior laxity [2,9].

In the acute post-injury phase, rigid braces have traditionally been recommended to limit posterior tibial translation to allow ligament healing [10,11]. Moreover, prolonged immobilization in knee extension is associated with complications including joint stiffness, atrophy of quadriceps muscle and delayed functional recovery [11,12].

Dynamic braces that apply a posterior to anterior directed force on the tibia, counteracting posterior translation during flexion, may offer a more effective and functional alternative for the conservative treatment of acute PCL injuries [11,13]. The positive roles of this brace have been demonstrated in cadaveric models, but no clinical studies have been reported on its effectiveness in posterior tibial translation reduction [10,14].

The aim of this study was to evaluate the effectiveness of a dynamic brace with posterior tibial support in reducing posterior tibial translation in acute PCL lesions. We tested four conditions: static (resting) = without brace and without anterior–posterior tibial stress (APs); AP stress = without brace but with APs; APs brace unloaded = wearing knee brace with APs but without posterior calf pad loaded; APs brace loaded = wearing knee brace with APs with posterior calf pad loaded.

## 2. Materials and Methods

A prospective study was performed at Papa Giovanni XXIII Hospital (Bergamo–Italy) on 11 patients, 9 male and 2 female, with a mean age of 29 years (range 18–56) with acute PCL injuries (4 isolated and 7 multiligament: 4 posteromedial and 3 posterolateral corner) included from July 2024 to June 2025. Diagnosis of PCL injury was established by physical examination and (magnetic resonance imaging) MRI scans. Inclusion criteria were: an acute lesion (within 15 days from trauma), a grade II (Hughston classification) or a higher PCL lesion grade with no associated fractures.

**Figure 1:**
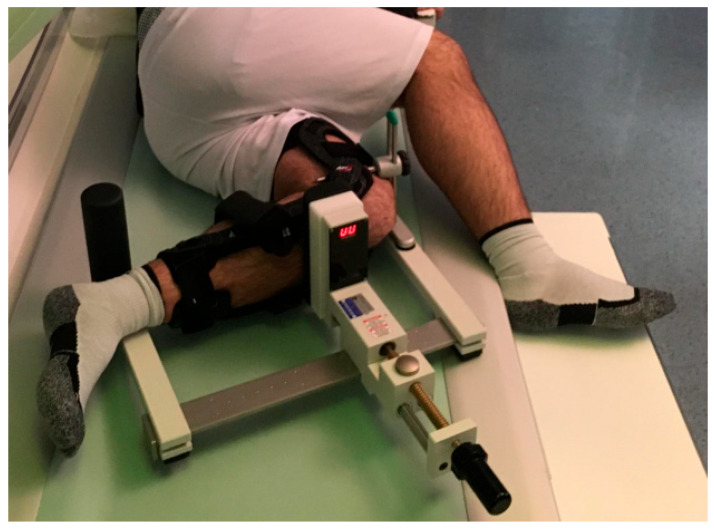
Patient position during stress radiography; with the knee in 90° flexion, a posterior stress radiograph was performed with use of the Telos device.

A standard lateral X-ray view and stress radiographs were performed in all patients by a single experienced operator (G.Z.) using a standardized method: knee at 90° flexion and a posteriorly directed force applied at the tibial tubercle level using the Telos device (Metax GmbH, Hungen-Obbornhofen, Germany) (Figure 1). A M.4^®^s PCL Dynamic brace (FGP, Dossobuono (VR)–Italy) was tested. The M.4^®^s PCL Dynamic brace is composed of a femoral and a tibial leg frame. The principle of four-chain linkage allows a constrained knee configuration or a free flexion and extension knee configuration allowing joint mobility while applying an adjustable anteriorly directed force to the calf [13].

**Figure 2:**
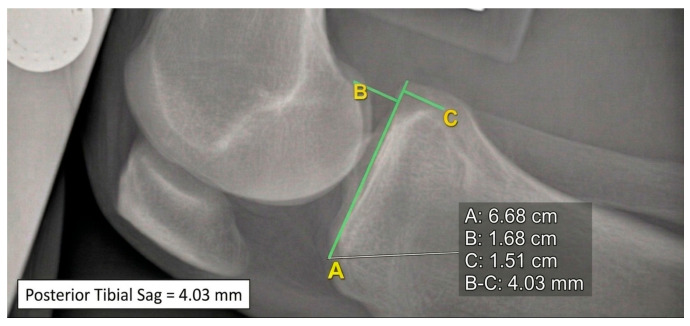
90° knee flexion without brace and without anterior–posterior tibial stress; posterior tibial translation = 4.03 mm.

**Figure 3:**
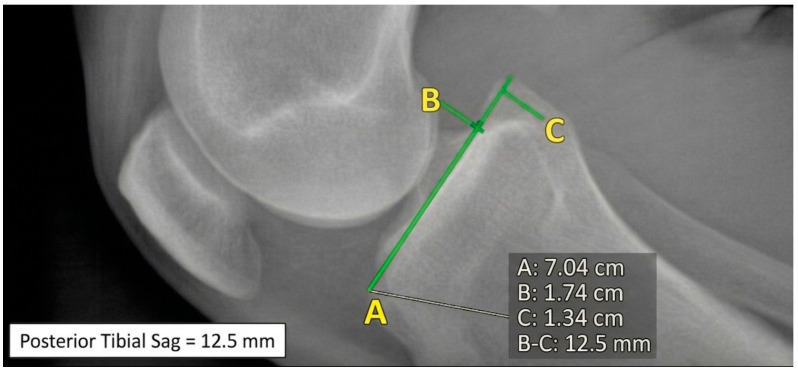
90° knee flexion without brace and anterior–posterior tibial stress; posterior tibial translation = 12.5 mm.

**Figure 4:**
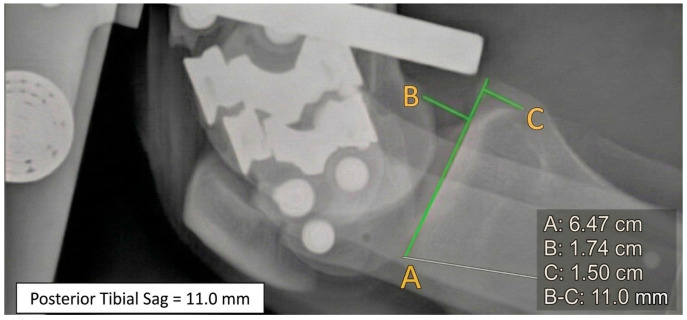
90° knee flexion with brace unloaded and anterior–posterior tibial stress; posterior tibial translation = 11 mm.

**Figure 5:**
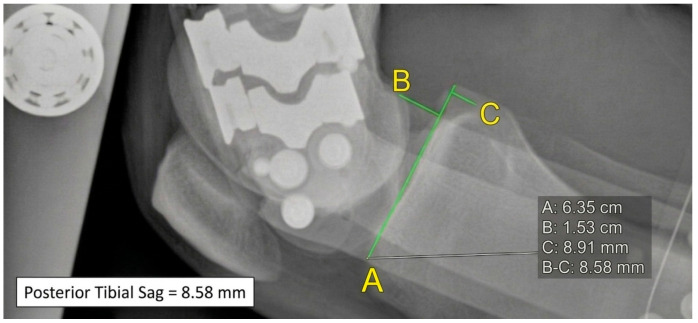
90° knee flexion with brace loaded and anterior–posterior tibial stress; posterior tibial translation = 8.58 mm.

The images were achieved in 4 four conditions:

**Static (resting)** = without brace and without anterior–posterior tibial stress (APs) (Figure 2);

**AP stress** = without brace but with APs (150 N) [13,14,15] (Figure 3);

**APs brace unloaded** = wearing knee brace with APs but without posterior calf pad loaded (Figure 4);

**APs brace loaded** = wearing knee brace with APs with posterior calf pad loaded (Figure 5).

In order to obtain the same amount of force applied to the calf for any patients, a sensor device (Sensor 5101, Tekscan Inc., South Boston, MA, USA) was placed between the posterior pad and the skin. The posterior pad was loaded to reach 50 N of posterior to anterior force [13]

Three orthopedic surgeons (M.D. N.T. C.P.), with different degrees of experience, performed the measurements of the posterior tibial displacement on all images according to the method described by Schulz et al. [14]; posterior tibial displacement was assessed by drawing a tangential line along the tibial plateau and perpendicular reference lines through the midpoints of the posterior contours of the femoral condyles and tibial plateau. The distance between these reference points represented tibial translation expressed in millimeters. Medial and lateral compartments were not analyzed separately.

The surgeons were blinded to the presence of the tibial stress and the loaded tibial support; the anterior tibial pad and posterior calf pad load were digitally excluded.

A power analysis was conducted, and it calculated an effect size (Cohen’s d) of 0.77 (2.0 mm divided by 2.6 mm). After setting an alpha value of 0.05, a one-tail paired *t*-test required 12 patients to obtain a power of 0.8 (R ver. 4.4.0 with RStudio ver. 2023.09 with pwr package ver. 1.3-0–function pwr.t.test).

A paired *t*-test, a Pearson correlation coefficient and the Levene test were used (C.I. 95%—*p* < 0.05). All data analyses were performed using IBM SPSS 28 Inc. (Armonk, N.Y., USA).

## 3. Results

**Figure 6:**
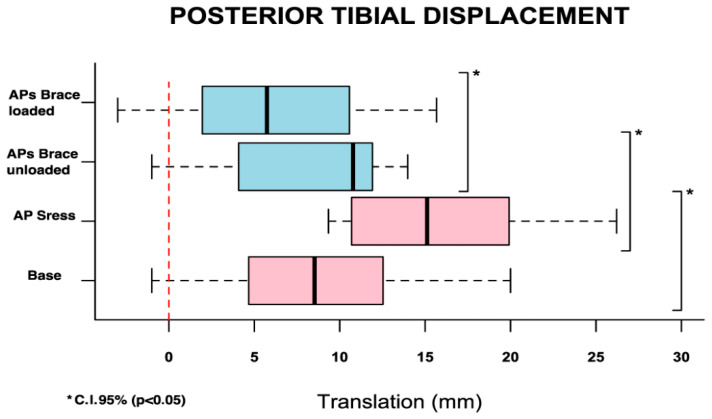
Posterior tibial displacement (C.I.: Confidence Interval). Boxes represent the confidence intervals, the vertical lines within the boxes indicate the medians, and the dashed lines denote the range of values. Pink: test without brace; blue: test with brace.

The patients were evaluated within 15 days from trauma (range, 5–13, median 8.9 days). The mean of the differences between “Static” and “AP stress” measure conditions indicate the whole knee posterior translation and was calculated at 7.1 ± 5.7 mm (*p* < 0.001) (Figure 6). The mean of the differences between “Static” and “APs brace unloaded” measure conditions show the static control of the knee with the brace and was calculated at 2.68 ± 3.4 mm (*p* < 0.027) (Figure 6). A positive linear correlation between these measures was observed (r = 0.856). The mean of the differences between “AP stress” and “APs brace loaded” measure conditions define the ability of the brace in reducing tibial posterior displacement during antero-posterior stress conditions (dynamic control) and was calculated at 6.81 ± 4.3 mm in favor of “APs brace loaded” (*p* < 0.001) (Figure 6) (see Supplementary Materials for individual test results). A positive linear correlation between these results was observed (r = 0.834). The Levene test found variance homogeneity of measures reported by the three surgeons in each condition and showed the reproducibility of the method despite surgeon experience.

## 4. Discussion

Conservative management of acute PCL lesions can yield satisfactory clinical long-term results if posterior knee stability is maintained [13]. Previous studies have emphasized that the prolonged use of a rigid brace or cast immobilization after PCL injury may cause knee joint stiffness and muscle atrophy leading to impaired knee function [11,12,14]. A dynamic knee brace can overcome these short- and medium-term complications allowing up to 90° knee flexion while applying posterior to anterior force on the tibia [4,12]. This function improves femoral tibial congruency, as shown by Heinrichs et al. [13], allowing an earlier return to and more physiological ambulation. Rasmussen et al. and Ahn et al. [6,7] demonstrated that flexion-assisted bracing may yield better clinical and imaging outcomes than immobilization alone. Compared to a straight brace, a dynamic brace is better tolerated by patients: poor tolerance and high dropout rates were reported for rigid braces due to discomfort and reduced quality of life [16]. Increased and more comfortable use, improving treatment adherence, has been reported [7]. Thus, this new approach appears to be a viable conservative option for acute PCL injuries, with the potential to improve short-term biomechanical outcomes and prevent chronic instability.

This study was focused on the use of a dynamic brace applying a posterior tibial force for the conservative management of acute PCL injuries, aiming to reduce posterior tibial translation. All patients included in the study had acute injuries diagnosed and evaluated within 15 days from trauma, a time window during which no ligament healing had occurred, avoiding potential bias derived from partial tissue healing during testing [5]. For objective assessment of posterior tibial translation, the Telos device was used since it is identified as the most sensitive tool for detecting PCL instability and minimizing rotational artifacts [15]. This setup ensured accurate and reproducible measurements. A 150 N of anterior to posterior applied force was chosen as suggested by previous studies [13,14,15]. Heinrichs et al. [13] assumed that patients might not be able to tolerate that amount of force being applied to the calf for a prolonged period of time. The posterior brace pad loading of 50 N was chosen based on biomechanical evidence suggesting that an applied brace with an anteriorly directed force of 50 N was necessary in order to restore posterior tibial translation to physiological values Heinrichs et al. [13]. Although the ideal loading threshold value for PCL failure is not defined in the literature, this experimental protocol resulted in effective testing in the behavior of the dynamic brace.

The strengths of the study are: All tests were conducted within 15 days from trauma, ensuring optimal evaluation during the healing window. A standardized protocol was applied for all patients by a single experienced operator: stress radiographs were performed using a validated device (Telos) and a sensor device which confirmed that the posterior pad was loaded to reach 50 N. Three blinded orthopedic surgeons, with different degrees of experience, performed the measurements with a high inter-observer reliability and a lower bias risk.

The weaknesses of this study are the relatively small number of patients and the heterogeneity of lesions: 11 patients, seven of whom presented multiligamentous injuries. However, due to the low incidence of PCL tears and isolated PCL injuries, it is quite difficult for a single center to collect a large amount of isolated acute PCL lesions.

Based on literature about the topic, to our knowledge no consensus about a recognized MCID (minimum clinical important difference) exists, which makes it difficult to conduct a power analysis. However, from a recent study by Brown et al. [17], it seems that 3.7 mm in posterior tibial displacement (PTT) for a patient affected by a PCL injury acts a threshold between “good” outcomes and worse symptoms. Another study [18] provided us with the mean and standard deviation for PTT in this kind of patient, which appeared to be 5.7  ±  2.6 mm. Given that it is reasonable to search for an effect size superior to the distance between the mean and the threshold, we calculated an effect size (Cohen’s d) of 0.77 (2.0 mm divided by 2.6 mm). A post hoc test with a sample size of 11 patients with the same parameters returned a power of 0.767, which we reasonably think is sufficient to draw our conclusions. The confidence intervals are wide because of a limited sample size, which, however, is very common in this field of research. However, for these reasons we expressed caution in generalizing our results.

A weight-bearing kinematic evaluation, such as a gait and dynamic knee motion analysis, was not carried out due to the limited research fundings.

To the best of our knowledge this is the first study that tests the reliability of a dynamic brace under loading on patients with acute PCL injuries. The aim was to evaluate only the effectiveness of a dynamic brace with posterior tibial support in reducing posterior tibial translation in acute PCL lesions. Clinical evaluation in terms of functional recovery, knee stability, and return to sport activities were not considered since it was beyond the objectives of our study.

Future studies on this topic may include a kinematic analysis to study biomechanical behavior of the knee with the dynamic brace, medium- and long-term patient reported outcome measures (e.g., IKDC, Lysholm, KOOS) to correlate with stress-radiographic findings, and a randomized prospective study comparing the dynamic knee brace with a straight brace in conservative treatment of acute PCL lesions.

## 5. Conclusions

This study demonstrates that the dynamic brace provides effective biomechanical reduction in posterior tibial translation in acute PCL injuries. These findings support the potential role of dynamic bracing in conservative treatment protocols.

## Data Availability

The original contributions presented in this study are included in the article/Supplementary Materials. Further inquiries can be directed to the corresponding author.

## Supplementary Materials

The following supporting information can be downloaded at: https://www.mdpi.com/article/10.3390/healthcare14070953/s1, Table S1. Results of individual patient tests according to the evaluations of the three different operators.

## References

[1] Tedeschi R., Giorgi F., Platano D., Berti L., Vita F., Donati D. (2025). Balancing Stability and Recovery: A Scoping Review on Conservative vs. Surgical Management of Acute Posterior Cruciate Ligament Injuries. Surgeries.

[2] Longo U.G., Viganò M., Candela V., de Girolamo L., Cella E., Thiebat G., Salvatore G., Ciccozzi M., Denaro V. (2021). Epidemiology of posterior cruciate ligament reconstructions in Italy: A 15-year study. J. Clin. Med..

[3] Sanders T.L., Pareek A., Barrett I.J., Kremers H.M., Bryan A.J., Stuart M.J., Levy B.A., Krych A.J. (2017). Incidence and long-term follow-up of isolated posterior cruciate ligament tears. Knee Surg. Sports Traumatol. Arthrosc..

[4] Barastegui D., Alentorn-Geli E., Gotecha D., Rius M., Navarro J., Cuscó X., Seijas R., Cugat R. (2021). Treatment of Partial Posterior Cruciate Ligament Injuries with Platelet-Rich Plasma in Growth Factors (PRGF) Intraligamentous Infiltration and a Specific Knee Brace. Surg. J..

[5] Vaquero-Picado A., Rodríguez-Merchán E.C. (2017). Isolated posterior cruciate ligament tears: An update of management. EFORT Open Rev..

[6] Rasmussen R.G., Jacobsen J.S., Blaabjerg B., Nielsen T.G., Miller L.L., Lind M. (2023). Patient-reported Outcomes and Muscle Strength after a Physiotherapy-led Exercise and Support Brace Intervention in Patients with Acute Injury of the Posterior Cruciate Ligament: A Two-year Follow-up Study. Int. J. Sports Phys. Ther..

[7] Ahn J.H., Lee S.H., Choi S.H., Wang J.H., Jang S.W. (2011). Evaluation of clinical and magnetic resonance imaging results after treatment with casting and bracing for the acutely injured posterior cruciate ligament. Arthrosc. J. Arthrosc. Relat. Surg..

[8] Jansson K.S., Costello K.E., O’Brien L., Wijdicks C.A., LaPrade R.F. (2013). A historical perspective of PCL bracing. Knee Surg. Sports Traumatol. Arthrosc..

[9] Gopinatth V., Mameri E.S., Casanova F.J., Khan Z.A., Jackson G.R., McCormick J.R., Brophy R.H., Knapik D.M., LaPrade R.F., Chahla J. (2023). Systematic Review and Meta-analysis of Clinical Outcomes After Management of Posterior Cruciate Ligament Tibial Avulsion Fractures. Orthop. J. Sports. Med..

[10] Li B., Shen P., Wang J.S., Wang G., He M., Bai L. (2015). Therapeutic effects of tibial support braces on posterior stability after posterior cruciate ligament reconstruction with autogenous hamstring tendon graft. Eur. J. Phys. Rehabil. Med..

[11] Jung Y.B., Tae S.K., Lee Y.S., Jung H.J., Nam C.H., Park S.J. (2008). Active non-operative treatment of acute isolated posterior cruciate ligament injury with cylinder cast immobilization. Knee Surg. Sports Traumatol. Arthrosc..

[12] Rasmussen R.G., Blaabjerg B., Nielsen T.G., Lind M. (2025). Long-term Follow-up of Patients with Acute Posterior Cruciate Ligament Injury Treated Non-operatively with a Physiotherapy-led Exercise and Support Brace Intervention. Int. J. Sports Phys. Ther..

[13] Heinrichs C.H., Schmoelz W., Mayr R., Keiler A., Schöttle P.B., Attal R. (2016). Biomechanical evaluation of a novel dynamic posterior cruciate ligament brace. Clin. Biomech..

[14] Schulz M.S., Steenlage E.S., Russe K., Strobel M.J. (2007). Distribution of Posterior Tibial Displacement in Knees with Posterior Cruciate Ligament Tears. J. Bone Jt. Surg..

[15] Jung T.M., Reinhardt C., Scheffler S.U., Weiler A. (2006). Stress radiography to measure posterior cruciate ligament insufficiency: A comparison of five different techniques. Knee Surg. Sports Traumatol. Arthrosc..

[16] Squyer E., Stamper D.L., Hamilton D.T., Sabin J.A., Leopold S.S. (2013). Unloader knee braces for osteoarthritis: Do patients actually wear them?. Clin. Orthop. Relat. Res..

[17] Brown J.S., Mogianos K., Roemer F.W., Isacsson A., Kumm J., Frobell R., Olsson O., Englund M. (2023). Clinical, patient-reported, radiographic and magnetic resonance imaging findings 11 years after acute posterior cruciate ligament injury treated non-surgically. BMC Musculoskelet. Disord..

[18] Bohe O., Greve F., Höger S., Mehl J., Siebenlist S., Willinger L. (2024). Posteromedial corner injuries result in the same posterior translation as posterolateral corner injuries in PCL ruptures. J. Exp. Orthop..

